# TSSC3 represses self-renewal of osteosarcoma stem cells and Nanog expression by inhibiting the Src/Akt pathway

**DOI:** 10.18632/oncotarget.20429

**Published:** 2017-08-24

**Authors:** Guang-Ning Yan, Xue-Feng Tang, Xian-Chao Zhang, Ting He, Yu-Sheng Huang, Xi Zhang, Gang Meng, De-Yu Guo, Yang-Fan Lv, Qiao-Nan Guo

**Affiliations:** ^1^ Department of Pathology, Xinqiao Hospital, Third Military Medical University, Chongqing 400038, PR China; ^2^ Institute of Pathology, Southwest Hospital, Third Military Medical University, Chongqing 400038, PR China; ^3^ Institute of Burn Research, Southwest Hospital, Third Military Medical University, Chongqing 400038, PR China

**Keywords:** TSSC3, Nanog, osteosarcoma, cancer stem cells, SRC

## Abstract

Osteosarcoma is the most common type of bone cancer, and the second leading cause of cancer-related death in children and young adults. Osteosarcoma stem cells are essential for osteosarcoma initiation, metastasis, chemoresistance and recurrence. In the present study, we report that: 1) higher TSSC3 expression indicates a better prognosis for osteosarcoma patients, and; 2) overexpression of TSSC3 significantly decreases sphere-forming capacity, tumor initiation, stemness-related surface markers and Nanog expression in osteosarcoma cells. We also discovered that higher Nanog expression correlates to a worse prognosis for osteosarcoma patients, and overexpression of Nanog increases the stem-related phenotype in osteosarcoma cells. Knockdown of Nanog suppresses these phenotypes. Inhibition of Nanog expression and self-renewal of osteosarcoma cells by TSSC3 overexpression appears to be mediated through inactivation of the Src/Akt pathway. In the clinical setting, expression of TSSC3, p-Src and Nanog is associated with recurrence, metastasis and surgical intervention. Lower TSSC3 expression, higher Nanog expression or higher p-Src expression indicate a poor prognosis for osteosarcoma patients. Overall, our study demonstrates that TSSC3 inhibits the stem-like phenotype and Nanog expression by inactivation of the Src/Akt pathway; this emphasizes the importance of Nanog in osteosarcoma stem cells.

## INTRODUCTION

Osteosarcoma (OS) is the most common type of bone cancer, and the fifth most common malignancy among individuals aged 15 to 19 years [1, 2]. Following a high dose of chemotherapy, chemoresistance is often induced and results in tumor recurrence [3]. The existence of osteosarcoma stem cells (OSCs) was first documented by Gibbs and colleagues, and has been confirmed in multiple OS cell lines by various means [4–6]. OSCs are considered to be responsible for OS initiation, metastasis, chemo-resistance and recurrence, have drawn a significant amount of attention [7]. Compared with differentiated cells, OSCs display stem-like phenotypes such as self-renewal, sphere formation ability, chemoresistance, and stem-related gene expression [8–12]. A more detailed understanding of OSCs is needed to develop targeted therapy and to improve prognosis.

TSSC3 (tumor-suppressing STF cDNA 3, also known as PHLDA2) was the first apoptosis-related gene reported to be imprinted, which is associated with growth inhibition and induction of apoptosis in human OS [13]. Our previous studies revealed that TSSC3 suppresses OS cell growth and inducing apoptosis [14]. Furthermore, we showed that it can reduce sphere formation and the volume of xenograft generated by OSCs [15]. Whether or not TSSC3 suppresses self-renewal of OSCs remains unclear.

Nanog is a gene involved in the pluripotency of embryonic stem cells, and has been reported to be an essential cancer stem cell transcription factor in several types of tumors [16–18]. However, little is known about the function of Nanog in OSCs. A previous study by our group found that TSSC3 can inactivate Src in OS cells. Src is a non-receptor tyrosine kinase (nRTK) that activates the Akt pathway [19, 20]. The Akt pathway has been reported to increase Nanog expression and promote cell survival in other tumors [21]. Since TSSC3 can induce apoptosis in OS cells, we hypothesized that TSSC3 could affect Src/Akt pathway activity, further regulating Nanog expression.

In this study, we aimed to establish the role of TSSC3 and the mechanisms through which it regulates OSCs. Furthermore, we attempted to determine whether Nanog is essential for maintenance of self-renewal capacity in OSCs. Our study highlights the important role of Nanog in maintaining OSCs, and suggests that the imprinted gene TSSC3 could be a promising target in OSC therapy.

## RESULTS

### TSSC3 overexpression suppresses the appearance of an OSC-like phenotype in OS cells and Nanog expression

**Figure 1:**
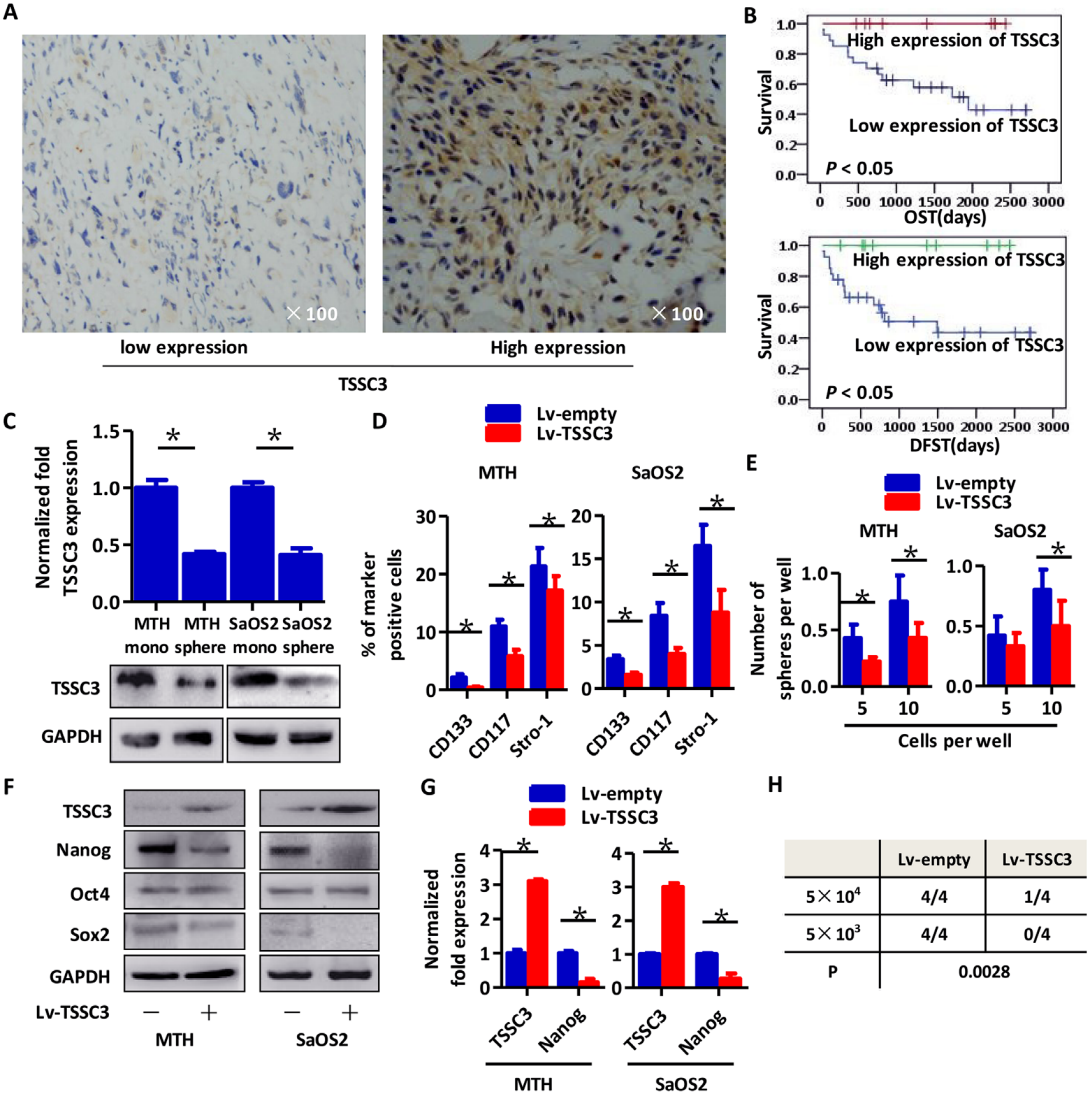
Higher expression of TSSC3 indicates improved prognosis for osteosarcoma patients, represses a stem-like phenotype of OS cells and decreases Nanog expression **(A)** Representative images of IHC staining of low (left panel) and high (right panel) TSSC3 expressing cells. **(B)** Kaplan-Meier curve showing that higher expression of TSSC3 is significantly associated with a better prognosis (P < 0.05). **(C)** TSSC3 expression in monolayer MTH or SaOS2 cells is higher than that of MTH or SaOS2 sphere cells, respectively (Bars, mean±SEM, *P < 0.05. Upper panel, RT-PCR; lower panel, Western blot). **(D)** The number of CD133, CD117, and Stro-1 positive MTH (left) or SaOS2 cells (right) significantly decreased after overexpression of TSSC3 (Bars, mean±SEM, *P < 0.05). **(E)** Efficiency of tumor sphere formation by Lv-TSSC3 MTH or Lv-TSSC3 SaOS2 cells is lower than that of Lv-empty MTH and SaOS2 cells, respectively (Bars, mean±SEM, *P < 0.05). **(F)** Nanog expression is reduced after TSSC3 overexpression in MTH (left) or SaOS2 (right) cells; meanwhile, Oct4 and Sox2 expression levels were slightly decreased. **(G)** Nanog expression is suppressed after overexpression of TSSC3 (Bars, mean±SEM, *P < 0.05). **(H)** There are significantly fewer xenografts generated by Lv-TSSC3 MTH than by Lv-empty MTH cells (N=4; *P* = 0.0028).

Representative images of TSSC3 on OS samples are shown in Figure 1A. Kaplan-Meier analysis revealed that expression of TSSC3 is positively associated with overall survival time (OST) and disease free survival time (DFST). Additionally, lower expression of TSSC3 indicated a poorer prognosis (Figure 1B). As shown in Figure 1C, TSSC3 expression decreases in MTH and SaOS2 sarcospheres compared with adherent monolayers of each respective cell type, at both mRNA and protein levels. To explore the effects of TSSC3 expression on sarcosphere formation, TSSC3 overexpression models (Lv-TSSC3) were established in both cell lines. FACS analysis demonstrated that the population of CD133+/CD117+/Stro-1+ OS cells was significantly decreased following TSSC3 overexpression (Figure 1D). Limiting dilution assays show that the sphere-forming capacity of TSSC3 overexpression cells is reduced compared to Lv-empty cells (Figure 1E). Following overexpression of TSSC3 in OS cells, Nanog expression was found to be down-regulated at both the protein (Figure 1F) and mRNA levels (Figure 1G). Meanwhile, there were no changes to the expression levels of Sox2 and Oct4. In the xenograft initiation assay, Lv-TSSC3 MTH cells generated less xenografts than Lv-empty MTH cells; this indicates that TSSC3 suppresses the tumor initiation ability of OS cells (Figure 1H). Since Nanog was reported to be an essential cancer stem cell transcription factor in several CSCs [25], it remains to be determine whether Nanog enhances the stem-like phenotype of OSCs.

### Overexpression of Nanog promotes a stem-like phenotype in Lv-TSSC3 OS cells

**Figure 2:**
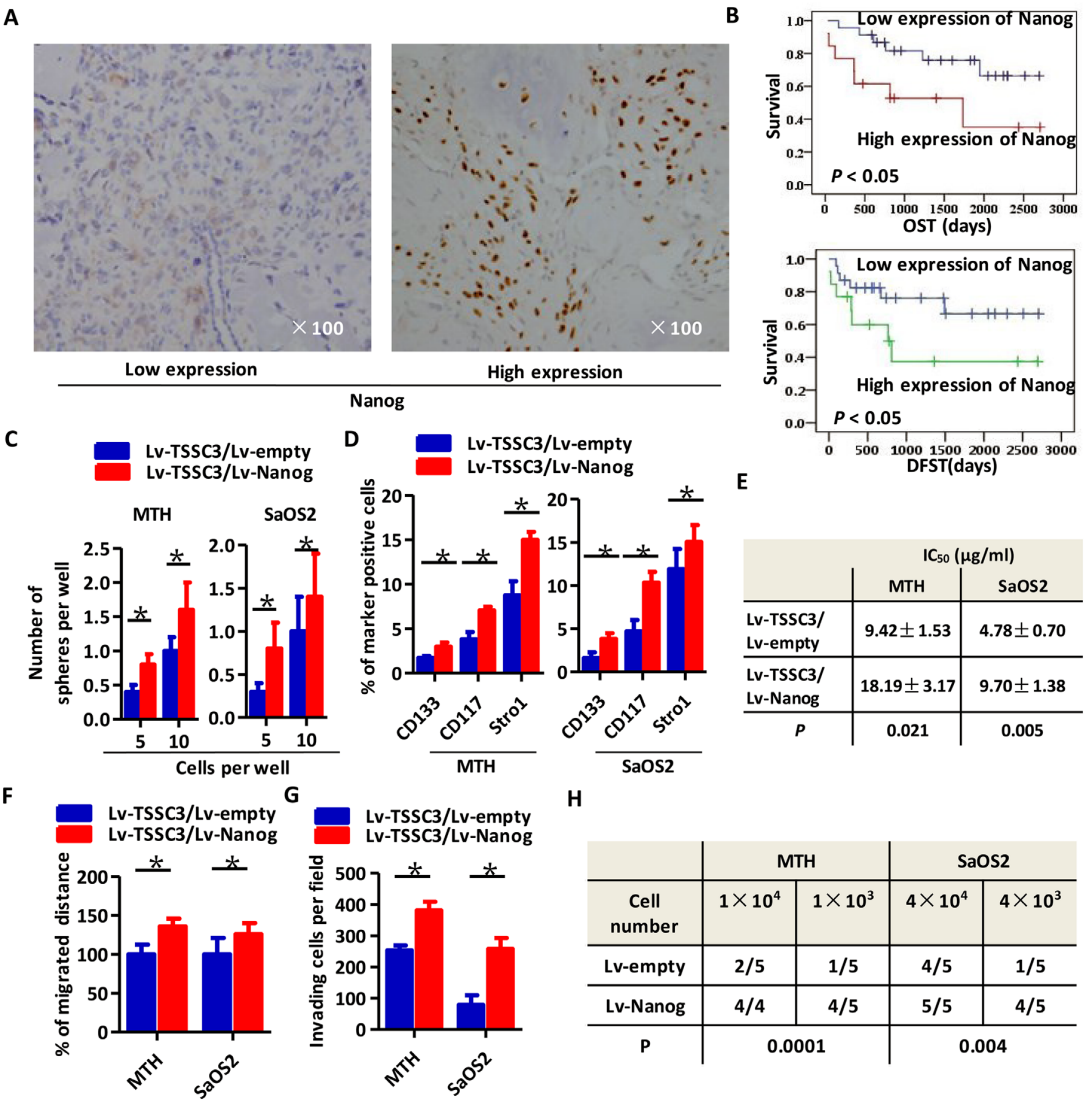
Higher expression of Nanog is associated with a worse prognosis for OS patients and significantly enhances the stem-like phenotype of OS cells **(A)** Representative IHC staining images of low (left panel) and high (right panel) Nanog expression. **(B)** Kaplan-Meier curve showing that higher expression of Nanog is significantly related to a poor prognosis (*P* < 0.05). **(C)** Efficiency of tumor sphere formation by Lv-TSSC3/Lv-Nanog MTH (left) or SaOS2 cells (right) is higher than in Lv-TSSC3/Lv-empty cells, respectively (Bars, mean±SEM, *P < 0.05). **(D)** The percentage of CD133, CD117 and Stro-1 positive Lv-TSSC3 MTH (left) or SaOS2 cells (right) is significantly increased after Nanog overexpression (Bars, mean±SEM, *P < 0.05). **(E)** The IC_50_ values of Lv-TSSC3 MTH and SaOS2 cells under cisplatin treatmentis are higher after Nanog overexpression. Migration **(F)** and invasion **(G)** capacity is enhanced in Lv-TSSC3 MTH and SaOS2 cells after Nanog overexpression (Bars, mean±SEM, *P < 0.05). **(H)** There are significantly more xenografts generated by MTH and SaOS2 cells after Nanog overexpression (N=5; *P =* 0.0001; *P =* 0.004).

Representative images of immunohistochemistry staining of OS clinical samples expressing Nanog are shown in Figure 2A. Kaplan-Meier analysis revealed that higher expression of Nanog was associated with a poorer prognosis of OST and DFST (Figure 2B). We established TSSC3 and Nanog overexpression cell models in MTH and SaOS2 cell lines (Lv-TSSC3/Lv-Nanog). Nanog overexpression results in markedly elevated expression of Oct4 and Sox2 (Supplementary Figure 1A). Overexpression of Nanog in Lv-TSSC3 MTH and SaOS2 cells significantly enhances sphere formation capacity, both in terms of efficiency (Figure 2C) and size (Supplementary Figure 1B). FACS analysis also reveals that overexpression of Nanog in Lv-TSSCs OS cells significantly increases the CD133+/CD117+/Stro-1+ population, compared with that of Lv-empty/Lv-TSSC3 OS cells (Figure 2D). The apoptosis assay showed that overexpression of TSSC3 increased the number of apoptotic cells both in MTH and SaOS2 cells compared with those from Lv-empty cells. Meanwhile, overexpression of Nanog decreased apoptotic cells compared with Lv-TSSC3/lv-empty cells (Supplementary Figure 1C & 1D). Nevertheless, the CCK-8 cell viability assay indicated that Lv-TSSC3/Lv-Nanog MTH and SaOS2 cells show greater resistance to cisplatin as compared to Lv-TSSC3/Lv-empty MTH and SaOS2 cells (Supplementary Figure 1E). The IC_50_ of Lv-TSSC3/Lv-Nanog MTH cells is 18.19 ± 3.17 μg/mL as compared to 9.42 ± 1.53 μg/mL in Lv-TSSC3/Lv-empty MTH cells (*P* < 0.05). The IC_50_ of Lv-TSSC3/Lv-Nanog SaOS2 cells is 9.70 ± 1.38 as compared to 4.78 ± 0.70 in Lv-TSSC3/Lv-empty SaOS2 cells (*P* < 0.05; Figure 2E). Migration assays (Figure 2F, Supplementary Figure 1F) and invasion assays (Figure 2G, Supplementary Figure 1G) confirmed that Nanog overexpression in Lv-TSSC3 MTH and SaOS2 cells could markedly improve cell motility. To determine the effect of Nanog on tumor initiation, subcutaneous xenograft models were generated. Overexpression of Nanog in Lv-TSSC3 MTH and SaOS2 cells significantly enhances tumor initiation (*P* < 0.05; Figure 2H, Supplementary Figure 1H). There were no significant differences between the volumes of xenografts generated by Lv-TSSC3/Lv-empty and Lv-TSSC3/Lv-Nanog OS cells (*P* > 0.05).

### Knockdown of Nanog expression reduces the stem-like phenotype in OS cells

**Figure 3:**
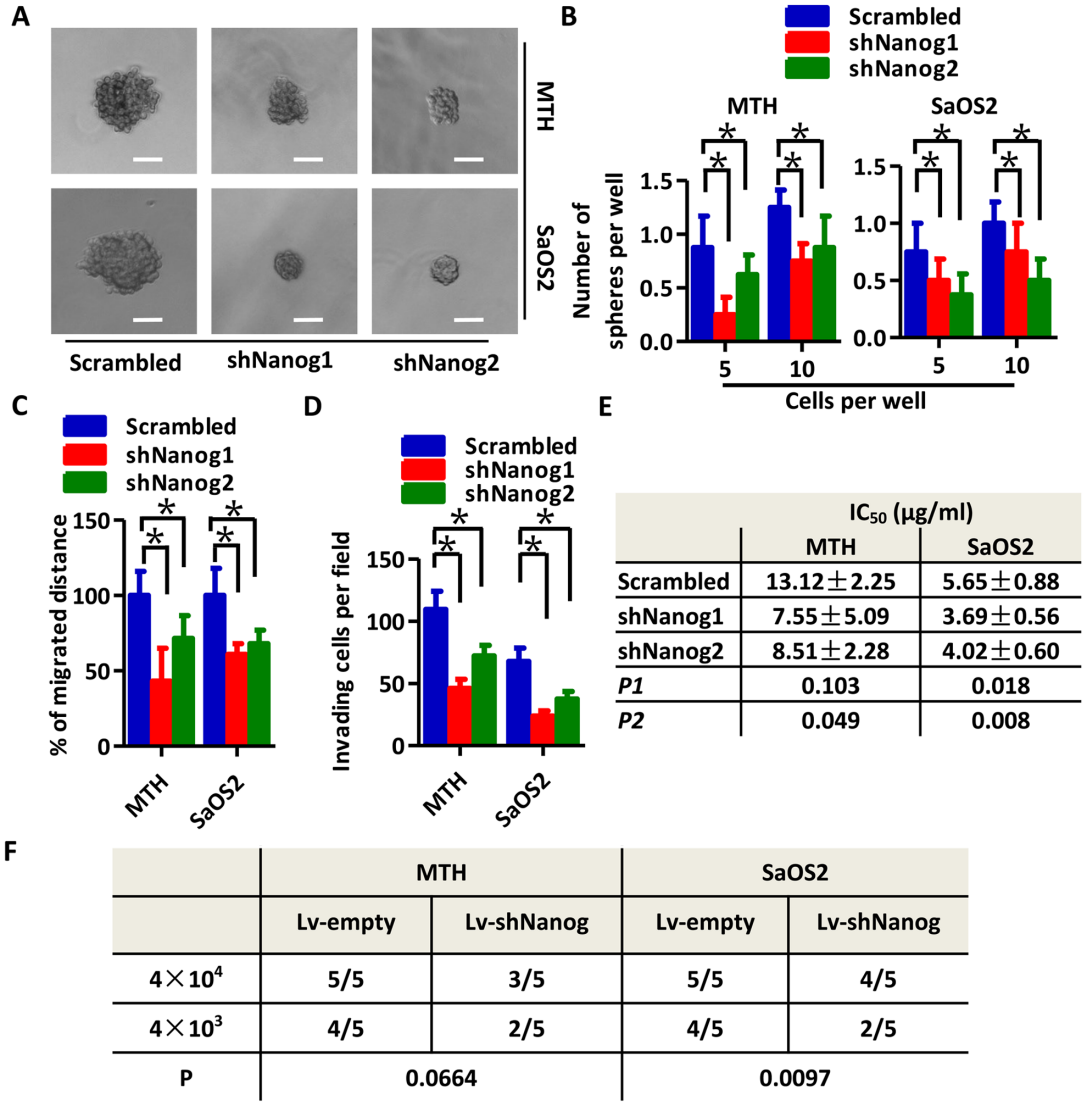
Knockdown of Nanog expression reduces the stem-like phenotype of OS cells **(A)** Tumor spheres formed by shNanog (shNanog1 and shNanog2) MTH (upper three images, the scale bar represents 100 μm) or SaOS2 cells (lower two images, the scale bar represents 100 μm) are smaller in size than those formed by scrambled MTH or SaOS2 cells, respectively. **(B)** Efficiency of tumor sphere formation by shNanog MTH (left) or SaOS2 cells (right) is lower than that by scrambled MTH and SaOS2 cells, respectively (Bars, mean±SEM, *P < 0.05). Migration ability **(C)** and invasion capacity **(D)** are reduced in shNanog MTH and SaOS2 cells as compared to their respective scrambled cells (Bars, mean±SEM, *P < 0.05). **(E)** The IC_50_ of shNanog1 (*P1*) and shNanog2 (*P2*) MTH and SaOS2 cells is lower than in scrambled cells treated with cisplatin. **(F)** There are fewer xenografts generated by Lv-shNanog SaOS2 cells than by Lv-empty SaOS2 cells (*P* = 0.0097); meanwhile, there are fewer generated by Lv-shNanog MTH cells (N=5; *P* = 0.0664).

To further examine the effects of Nanog on a stem-like phenotype in OS cells, we established shNanog-plasmid transfected OS cells (shNanog). The knockdown efficiency of two sequences (shNanog1 and shNanog2) were tested by Western blot (Supplementary Figure 2A) and immunofluorescence (Supplementary Figure 2B). As shown in Figure 3A, tumor spheres generated by shNanog MTH and SaOS2 cells are significantly smaller than those generated by scrambled shRNA cells. Results from the limited dilution assay are consistent with our hypothesis that knockdown of Nanog in OS cells inhibits sphere-forming ability (Figure 3B). In migration assays, Nanog knockdown markedly reduced cell motility (Figure 3C, Supplementary Figure 2C). Results of the invasion assay also confirm the aforementioned findings (Figure 3D, Supplementary Figure 2D). An apoptosis assay showed that knockdown of Nanog in MTH and SaOS2 cells decreased the number of apoptotic cells compared with those from scrambled cells (Supplementary Figure 2E). The does-response curves from the CCK-8 assay show reduced viability of Nanog knockdown OS cells following treatment with cisplatin (Supplementary Figure 2F). The IC_50_ of scrambled MTH cells is 13.12 ± 2.25 μg/mL as compared to 7.55 ± 5.09 μg/mL in shNanog1 MTH cells (*P* > 0.05), and 8.51 ± 2.28 μg/mL in shNanog2 MTH cells (*P* < 0.05). In SaOS2 cells, the IC_50_ is 5.65 ± 0.88 μg/mL in scrambled cells, 3.69 ± 0.56 μg/mL in shNanog1 cells (*P* < 0.05), and 4.02 ± 0.60 μg/mL in shNanog2 cells (*P* < 0.05; Figure 3F). Xenograft initiation by Lv-shNanog SaOS2 cells decreases as compared to Lv-empty cells, but not so significant in MTH cells; this requires further investigation (Figure 3F, Supplementary Figure 2G). Statistical analyses show that there are no significant differences between the volumes of xenografts generated by Lv-empty and Lv-shNanog OS cells (*P* > 0.05). Therefore, our data suggests that Nanog is very important in promoting a stem-like phenotype in OS cells.

### TSSC3 inhibits Src activity to suppress Nanog expression and self-renewal of OS cells

**Figure 4:**
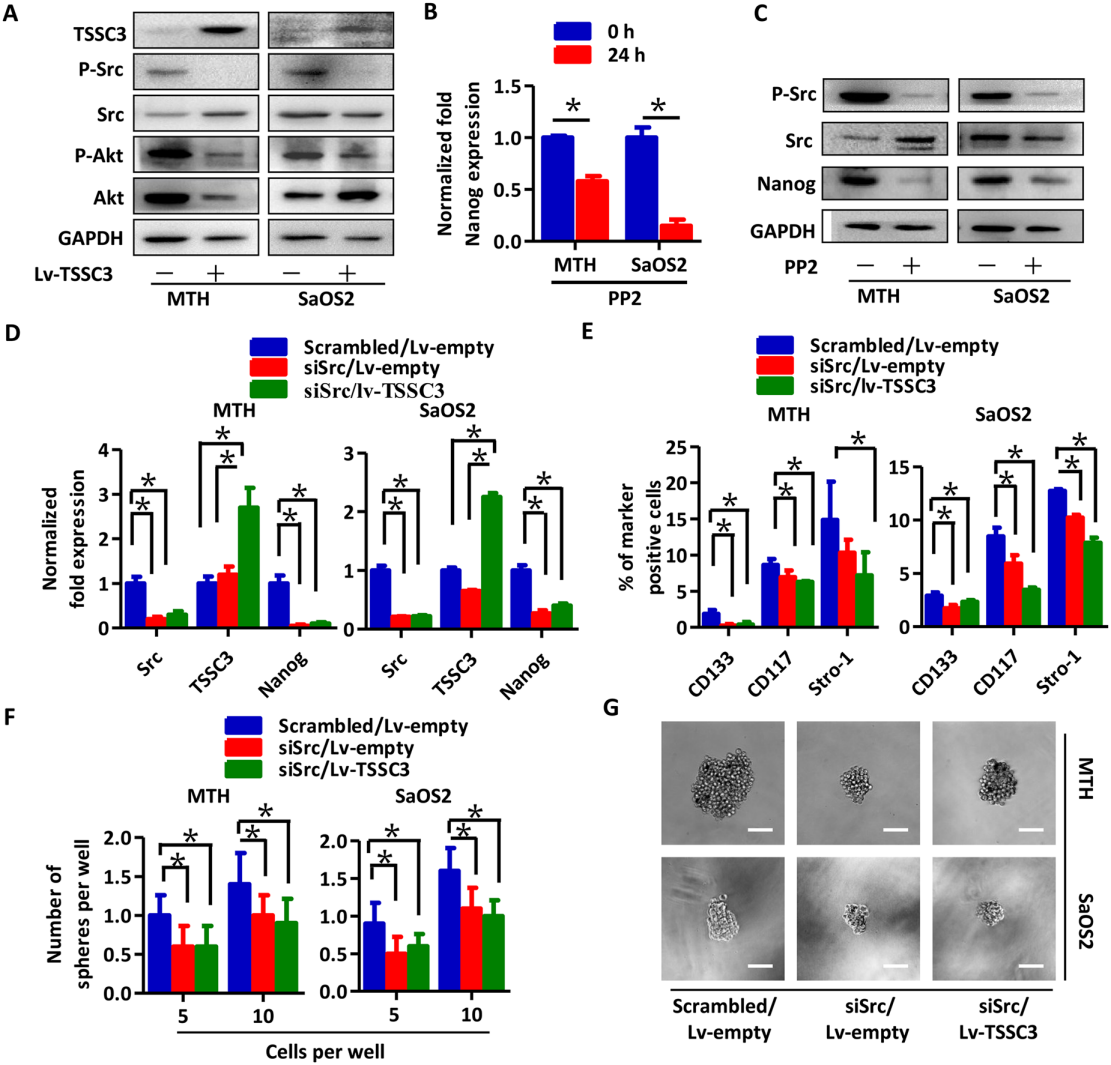
TSSC3 inhibition of Nanog expresssion is mediated by Src **(A)** Overexpression of TSSC3 inhibits Src/Akt pathway activity in OS cells. RT-PCR **(B)** and Western blot analysis **(C)** show that inactivation of Src results in reduced Nanog expression in OS cells (Bars, mean±SEM, *P < 0.05). **(D)** Knockdown of Src results in reduced Nanog expression in MTH (left) and SaOS2 cells (right; Bars, mean±SEM, *P < 0.05), while overexpression of TSSC3 does not further reduce Nanog expression (Bars, mean±SEM, *P* > 0.05). **(E)** The number of CD133, CD117 and Stro-1 positive MTH (left) and SaOS2 cells (right) is significantly decreased after Src knockdown (Bars, mean±SEM, *P < 0.05), while overexpression of TSSC3 only slightly further decreases expression of these markers. **(F)** Efficiency of tumor sphere formation by Lv-empty/siSrc MTH (left) and SaOS2 cells (right) is lower than that of scrambled MTH and SaOS2 cells (Bars, mean±SEM, *P < 0.05), while overexpression of TSSC3 does not have a significant additional influence on sphere formation. **(G)** Tumor spheres formed by Lv-empty/siSrc MTH (upper three images, the scale bar is 100 μm) or SaOS2 cells (lower three images, the scale bar is 100 μm) are smaller in size than those formed by scrambled/Lv-empty MTH or SaOS2. Overexpression of TSSC3 does not have a significant influence on the size of tumor spheres in siSrc cell lines.

After TSSC3 overexpression, p-Src and p-Akt levels decrease, indicating that TSSC3 suppresses Src activity (Figure 4A). Nanog expression was down-regulated following PP2 treatment (a Src inhibitor [26]), both at the level of mRNA (Figure 4B) and protein (Figure 4C), indicating that Src activity stimulated Nanog expression. Next, we used two siRNAs to establish Src knockdown cell models (siSrc; Supplementary Figure 3A & 3B). Src knockdown decreases levels of Nanog in MTH and SaOS2 cells (*P* < 0.05), while overexpression of TSSC3 in siSrc cells does not further inhibit Nanog expression (*P* = 0.116, MTH cells; *P* = 0.063, SaOS2 cells; Figure 4D). As examined by FACS, Src knockdown results in a decrease in the CD133/CD117/Stro-1 positive population of MTH and SaOS2 cells, and TSSC3 overexpression appears to further decrease this population, which may due to regulation of surface marker expression by TSSC3 through another mechanism independent of Src inhibition (Figure 4E). The sphere-forming assay also showed the same results in regard to both number (Figure 4F) and volume (Figure 4G). These data demonstrate that TSSC3 down-regulates Nanog expression via inhibition of Src activity.

### TSSC3 regulation of Nanog expression and self-renewal of OS cells is mediated through the Akt pathway

**Figure 5:**
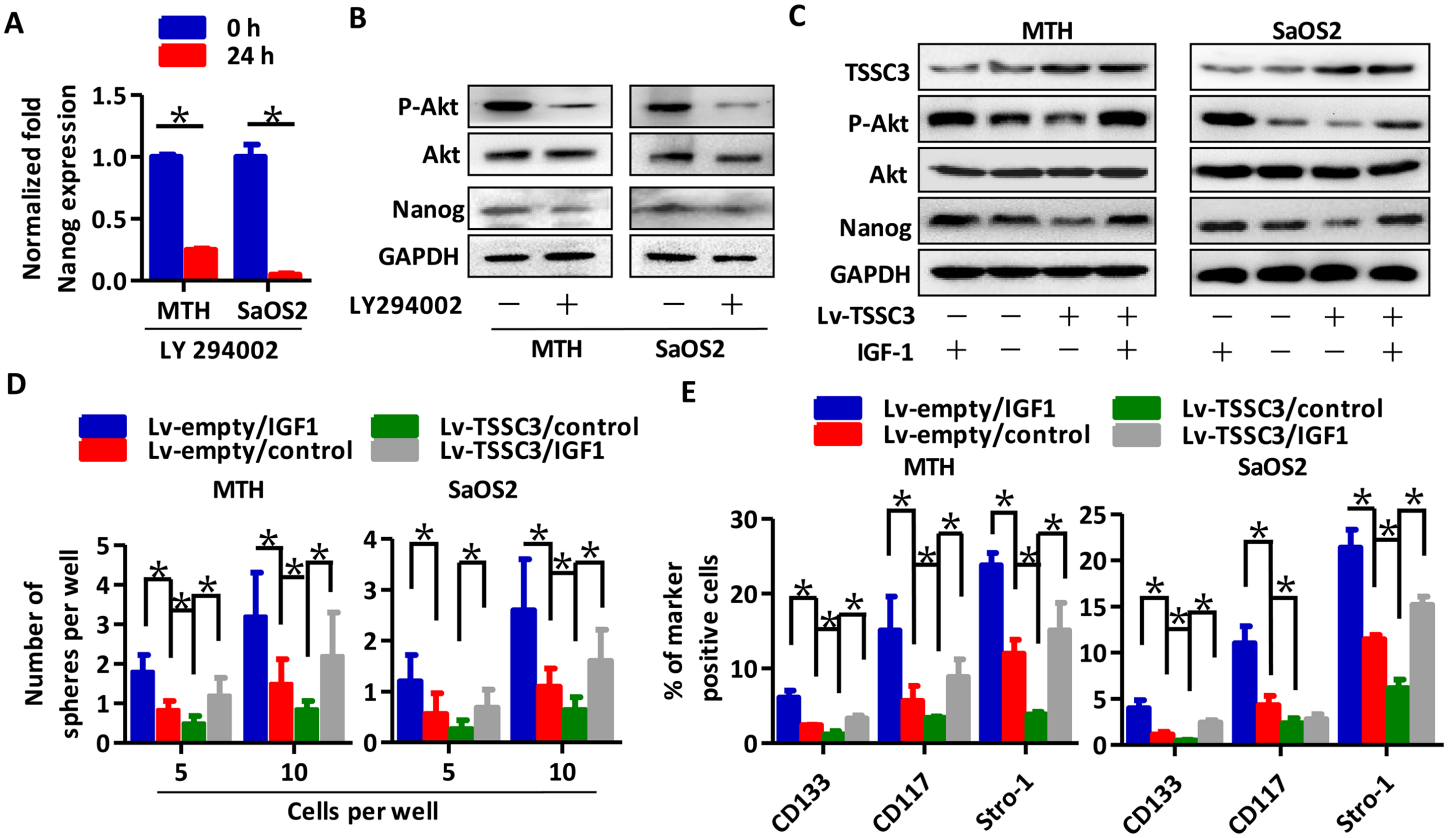
TSSC3 inhibits Nanog expression through the Akt pathway RT-PCR **(A)** and Western blot analysis **(B)** show that inactivation of Akt results in reduction of Nanog in OS cells (Bars, mean±SEM, *P < 0.05). **(C)** Overexpression of TSSC3 inactivates the Akt pathway, and decreases Nanog expression in MTH (left) and SaOS2 cells (right). Treatment with IGF1 activates the Akt pathway and increases Nanog expression. **(D)** Efficiency of tumor sphere formation by Lv-TSSC3 MTH (left) or SaOS2 cells (right) is lower than that of Lv-empty MTH and SaOS2 cells. IGF1 treatment enhances the efficiency (Bars, mean±SEM, *P < 0.05). **(E)** The number of CD133, CD117 and Stro-1 positive Lv-TSSC3 MTH (left) or SaOS2 cells (right) significantly decreases compared to Lv-empty cells. IGF1 treatment significantly enhances the number of marker positive cells (Bars, mean±SEM, *P < 0.05).

Levels of Nanog mRNA (Figure 5A) and protein (Figure 5B) were both downregulated following LY294002 treatment (an Akt inhibitor [27]); this suggests that the Akt pathway can stimulate Nanog expression. Next, we used IGF-1 for Akt pathway activation [28]. TSSC3 overexpression inhibits Akt activity, and reduces Nanog expression. Following IGF-1 treatment, the Akt pathway is significantly activated, and Nanog expression is markedly increased, both in Lv-empty and Lv-TSSC3 MTH and SaOS2 cells (Figure 5C). Moreover, the efficiency (Figure 5D) and volume (Supplementary Figure 3C) of spheres generated by Lv-TSSC3 cells is reduced, while IGF-1 treatment significantly increases efficiency and volume. FACS analysis also showed consistent results (Figure 5E). These data suggest that the Akt pathway mediates the regulation of Nanog expression and self-renewal of OS cells by TSSC3.

### Clinical and pathological significance of TSSC3, p-Src, p-Akt and Nanog expression in OS samples

**Figure 6:**
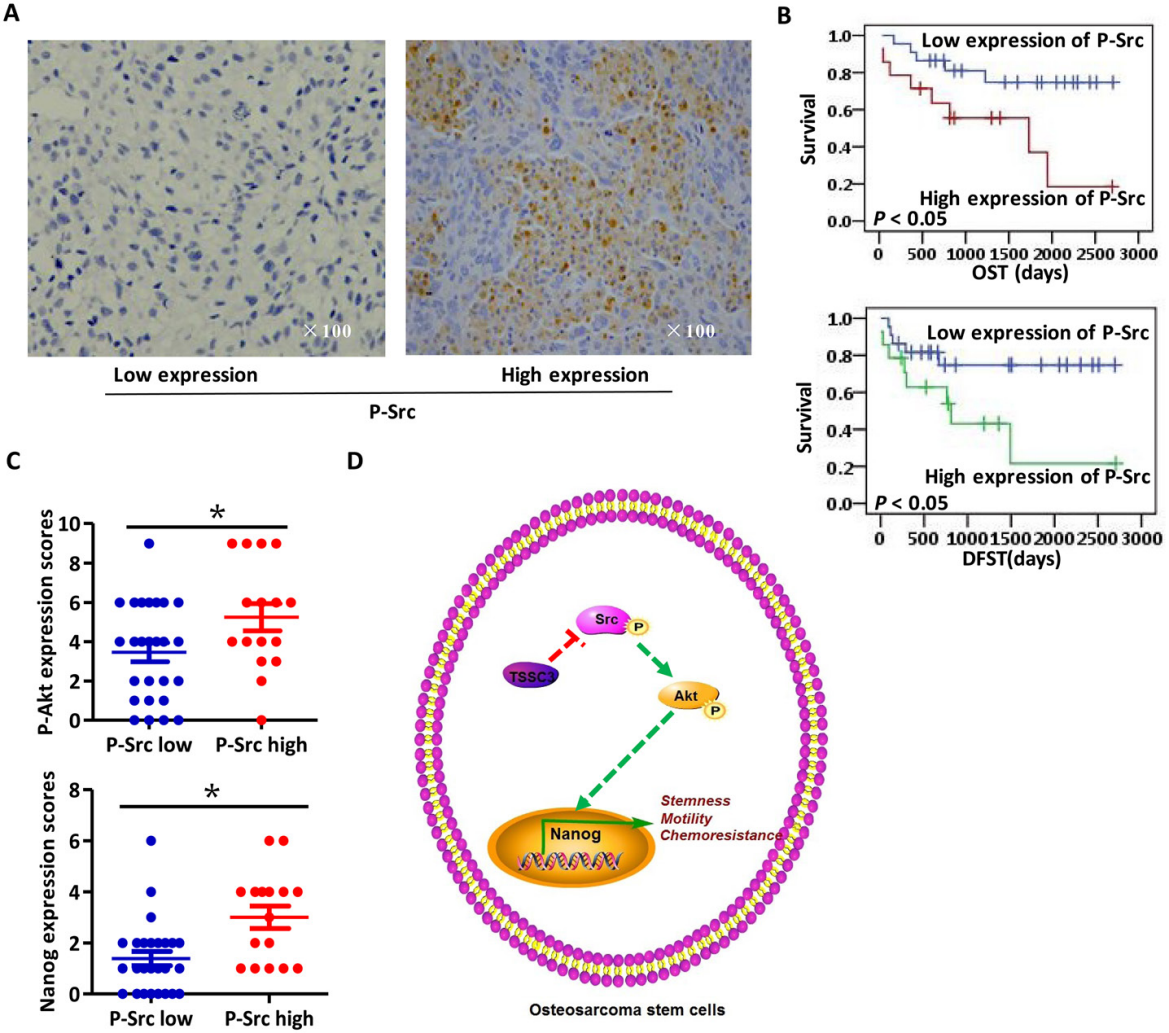
Clinical analysis of IHC staining of osteosarcoma samples **(A)** Representative images of IHC staining for low (left) and high (right) p-Src expression levels. **(B)** Kaplan-Meier curve showing that lower expression of p-Src is significantly associated with an improved prognosis (*P* < 0.05). **(C)** Scores of IHC staining for p-Src and p-Akt are positively associated (upper panel, Bars, mean±SEM, *P < 0.05); meanwhile, those for p-Src and Nanog are also positively associated (lower panel, Bars, mean±SEM, *P < 0.05). **(D)** Schematic summarizing our findings. In OS cells, higher Nanog expression significantly enhances stemness, motility and chemoresistance. TSSC3 inhibits Src activity, resulting in decreased activity of the Akt pathway and leading to a reduction in Nanog expression and stem-like phenotype in OS cells.

Representative images of p-Src expression in OS samples are shown in Figure 6A; images of p-Akt are shown in Supplementary Figure 3D. The percentage of cells expressing TSSC3, p-Akt, p-Src and Nanog in osteoblastic OS samples is 26.7% (8/30), 43.3% (13/30), 63.3% (19/30) and 40.0% (12/30), respectively. In chondroblastic OS samples, the percentage of cells expressing TSSC3, p-Akt, p-Src and Nanog is 12.5% (1/8), 12.5% (1/8), 75% (6/8) and 12.5% (1/8), respectively. In fibroblastic OS cells, the percentage of cells expressing TSSC3, P-Akt, P-Src and Nanog is 33.3% (1/3) in all cases. Kaplan-Meier analysis revealed that lower expression of p-Src indicates a better prognosis (Figure 6B). Expressions of TSSC3, p-Akt, p-Src and Nanog are not significantly associated with the parameters of age, clinical stage, tumor location and whether or not chemotherapy is used. Patients with local recurrence and male OS patients are more likely to have lower expression of TSSC3; patients with metastases are more likely to have higher expression of p-Src and Nanog; patients who underwent amputation are more likely to have higher p-Src expression (Table 1). In clinical samples, p-Src expression is associated with p-Akt and Nanog expression (Figure 6C). Taken together, we believe that these data demonstrate that TSSC3, p-Src and Nanog are important clinical markers to predict tumor progression and patient prognosis.

**Table 1:** Correlation between IHC staining and pathological parameters in osteosarcoma patients

Feature		TSSC3			p-Src			p-Akt			Nanog		
		Neg. (n)	Pos. (n)	*P*	Neg. (n)	Pos. (n)	*P*	Neg. (n)	Pos. (n)	*P*	Neg. (n)	Pos. (n)	*P*
Gender	Male	21	3	0.037	13	11	0.149	9	15	0.887	16	8	0.897
	Female	10	7		13	4		6	11		11	6	
Age	≤30	22	9	0.135	19	12	0.687	11	20	0.846	19	12	0.393
	>30	9	1		7	3		4	6		8	2	
Histological	Osteoblastic	22	8	0.598	17	13	0.281	11	19	0.451	18	12	0.355
type	Chondroblastic	7	1		7	1		2	6		7	1	
	Fibroblastic	2	1		2	1		2	1		2	1	
Stage	IIA	14	4	0.528	12	6	0.925	4	14	0.143	11	7	0.790
	IIB	10	5		9	6		6	9		10	5	
	III	7	1		5	3		5	3		6	2	
Recurrence	Yes	14	1	0.047	8	7	0.315	6	9	0.733	9	6	0.553
	No	17	9		18	8		9	17		18	8	
Metastasis	Yes	10	2	0.590	5	7	0.039	6	6	0.873	5	7	0.038
	No	21	8		21	8		9	20		22	7	
Surgery	Excision of lesion	15	6	0.528	17	4	0.018	9	12	0.399	15	6	0.446
	Amputation & other	16	4		9	11		6	14		12	8	
Chemotherapy	Yes	19	7	0.718	16	10	0.799	9	17	0.779	18	8	0.640
	No	12	3		10	5		5	9		9	6	

## DISCUSSION

Emerging evidences suggest that malignant tumors contain a hierarchy of cells responsible for tumor initiation, propagation, recurrence and resistance to therapy. These cancer stem cells (CSCs) have the ability to retain their stem cell-like properties through self-renewal and differentiation [29, 30]. OSCs were identified as a subpopulation of cells in human OS tissue samples and cell lines that were capable of forming sarcospheres in serum-free conditions, and are considered the “seeds” of OS initiation and recurrence [10, 31]. Therapies targeting OSCs are urgently needed, and may improve patient prognosis.

TSSC3, also known as PHLDA2, is an imprinted gene [32]. Genomic imprinting has been reported to contribute to tumorigenesis by predisposing individuals to cancer [33]; this is mainly regulated by epigenetics [34]. Loss of expression of TSSC3 has also been reported in malignant tumors [35]. Our lab has previously demonstrated that TSSC3 acts as a tumor suppressor gene due to its pro-apoptotic function, inhibition of cell growth and enhancement of sensitivity to chemotherapy in OS cells [14, 36], and MECP2 (methyl-CpG-binding protein 2) and EZH2 (Enhancer of zeste homolog 2) are involved in regulation of TSSC3 expression [22, 32, 37]. Here, we demonstrated that TSSC3 suppresses self-renewal in stem-like OS cells, including sphere formation ability, stem-related surface marker expression, stem-related nuclei marker expression and the ability to initiate xenograft formation. Clinically, we found that lower expression of TSSC3 was more likely to be associated with local recurrence and poor outcomes in OS patients. These findings provide a more detailed understanding of the importance of TSSC3 in tumor initiation and progression.

The transcription factors Oct3/4, Sox2, and Nanog have been used as nuclear markers to identify OSCs [38–40]. Oct3/4 and Sox2 were reported to be involved in maintenance and enhancement of self-renewal of OSCs, but less is known about the role of Nanog in maintaining OSCs [41–43]. Here, we report that overexpression of Nanog enhances Oct4 and Sox2 expression, sphere formation, stem-related surface marker expression, chemoresistance, migration, invasion and tumorigenicity in OS cells. Knockdown of Nanog inhibits these features. Clinically, higher expression of Nanog indicates a worse prognosis for patients and higher rates of metastasis. These results suggest that, in OSCs, Nanog is very important for maintenance and enhancement of self-renewal. Since the expression of Nanog is suppressed by TSSC3, the mechanism through which this occurs is worth exploring.

The upstream regulators of Nanog were reported to include the IGF pathway, p53, Akt pathway and several miRNAs [16, 17, 44]. In our research, we found that the Src pathway was involved in Nanog regulation in OS. Src was the first oncogene to be discovered with tyrosine kinase activity to activate the Akt pathway [45], and leads to anchorage-independent growth and anoikis resistance [46]. Studies from our lab indicate that TSSC3 binds to Src with Ranbp9 and prevents its phosphorylation, resulting in apoptosis [47], which prompted us to hypothesize that TSSC3 inhibition of Nanog expression was likely mediated by Src inactivation. We then demonstrated that overexpression of TSSC3 inactivates the Src/Akt pathway, and Src inactivation in OS cells inhibits Nanog expression. Knockdown of Src expression results in reduction of Nanog expression, stem-related surface marker expressions and sphere formation ability. Overexpression of TSSC3 in Src knockdown cells could not further suppress Nanog expression and self-renewal. In patients, activation of Src was associated with higher expression of p-Akt and Nanog, more metastasis, more amputation and a worse prognosis. These results indicate that Src mediates suppression of Nanog by TSSC3, and that this process is of clinical importance.

PHLDA3, a TSSC3 homolog, was reported be a repressor of the Akt pathway, which suggested to us that TSSC3 could have the same effect [48]. In lung cancer and embryonic carcinoma cells, the Akt pathway was reported to induce Nanog expression [21, 49], linking the TSSC3/Src/Akt/Nanog axis. In the present study, we identified the Akt pathway as a target of TSSC3, which regulates Nanog expression in OS. We found that after Akt inactivation, Nanog expression was notably decreased. When the Akt pathway was persistently activated, Nanog expression, sphere formation and stem-related surface marker expression were all greatly increased in OS cells, independent of TSSC3 overexpression. CD44 and CXCR4 expression were also tested in OS cells. Overexpression of TSSC3 reduced CD44 and CXCR4 expression in OS cells; meanwhile, overexpression of Nanog increased CD44 and CXCR4 expression in OS cells (Supplementary Figure 3E). IHC stainings of xenografts confirmed the TSSC3/Src/Akt/Nanog signaling pathway, as xenografts generated by TSSC3 overexpression cells showed lower Nanog, P-Src, and P-Akt expression levels. Overexpression or knockdown of Nanog did not regulate TSSC3 expression in xenografts (Supplementary Figure 4). Our data link Src and Akt to cancer stem cells, which further highlights the importance of TSSC3 and Nanog in OSCs.

There are certain limitations to our study. Src has multiple functions beyond activating the Akt pathway, including roles in the Ras-MAPK, FAK and STAT3/c-myc pathways [46]. The mechanisms involved in induction of Nanog expression by the Akt pathway are also numerous and require further exploration.

## MATERIALS AND METHODS

### Primary osteosarcoma samples

OS specimens were obtained from 41 patients who underwent surgery with no prior chemotherapy or radiation therapy at the Xinqiao Hospital, Third Military Medical University, Chongqing, between 2008 and 2015 (Supplementary Table 1). Among them, only patients who had surgery a year prior to our analysis were considered for prognostic analysis. All samples were identified as high grade OS by a senior pathologist. All experiments were carried out in accordance with the Declaration of Helsinki, and were approved by the Institutional Ethics Committee of Xinqiao Hospital, Third Military Medical University (No. 27, 2011). Written informed consent for the biological studies was obtained from the patients or their guardians.

### Immunohistochemistry

Immunohistochemistry (IHC) was performed using an IHC kit (ZSGB-bio, Beijing, China). The experiments were performed as previously described [22]. For human osteosarcoma specimens, primary mouse anti-human Nanog (1:100; Cell Signaling Technology, Danvers, MA), rabbit anti-human p-Src (1:100; Biodragon Immunotech, China), mouse anti-human TSSC3 (1:100; Novus biologicals, Littleton, CO), rabbit anti-human p-Akt (1:100; Biodragon Immunotech) and rabbit anti-human Ki67 (1:100; ZSGB-bio) were used. For xenograft specimens, primary rabbit anti-mouse Nanog (1:100; Cell Signaling Technology, Danvers, MA), rabbit anti-mouse p-Src (1:100; Cell Signaling Technology, Danvers, MA), rabbit anti-mouse TSSC3 (1:100; Biodragon Immunotech), and rabbit anti-human p-Akt (1:100; Cell Signaling Technology, Danvers, MA) were used. Secondary antibodies included horseradish peroxidase (HRP)-conjugated goat anti-mouse IgG and goat anti-rabbit IgG. Specimens were independently scored by two pathologists who were blinded to the clinical and pathological data. For semi-quantitative assessment of protein expression, the percentage of positive cells was calculated in more than five randomly selected fields of view with higher-magnification objectives, and included over 100 cells. The final score is a product of the positive cell ratio score (0, 0–10% positive; 1, 10–50% positive; 2, 50–80% positive; 3, > 80% positive) and relative expression score (1, yellow staining; 2, brown staining; 3, dark brown staining). Final scores ≥ 3 were considered positive [22].

### Cell culture

The human OS cell line SaOS2, immortalized human osteoblasts, and hFOBs (hFOB1.19) were obtained from American Type Culture Collection (ATCC, Manassas, VA). The malignant transformed hFOB1.19 cell line (MTH) was accomplished in our lab by sequential treatment of the initiator N-methyl-N’-nitro-N-nitrosoguanidine (MNNG), and a promoter, 12-O-tetradecanoylphorbol-13-acetate (TPA), as described previously [23]. Cells were cultured using Dulbecco's Modified Eagle medium (DMEM), containing 10% fetal bovine serum (FBS) at 37 °C. Tumor spheres were cultured in 6-well ultra-low attachment plates (Corning, Tewksbury, MA) in stem cell medium which consisted of serum-free DMEM/F12 medium with 20 ng/mL epidermal growth factor (EGF; PeproTech, Rochy Hill, NJ), 20 ng/mL bFGF (PeproTech), and B27 (1×, Sigma-Aldrich, St. Louis, MO). Medium was changed every two days.

### Gene knockdown and overexpression experiments

The mRNA sequences of human TSSC3 (NM_003311.3), Nanog (NM_176996.4) and SRC (NM_005417.4) were acquired from the NCBI database. A lentivirus expression vector (pLVX-Puro) encoding TSSC3 and an empty vector control were purchased from Sangon Biotech Co. (Guangzhou, China). A shRNA-lentivirus vector (pLVX-shRNA1) encoding Nanog and an empty vector control were purchased from Biodragon Immunotech Co. (Beijing, China). Both of the above-mentioned lentiviral vectors were prepared using the Lenti-X HT Packaging System (Clonetech, Mountain View, CA). The lentivirus expression vector (pLOV-EF1a-PuroR-CMV-eGFP-3FLAG) encoding Nanog and the empty vector control were synthesized using the ViraPower Lentiviral Expression System (Invitrogen, Carlsbad, CA) and purchased from Neuron Biotech Co. (Shanghai, China). Infection protocols were performed according to the instructions from the lentiviral kits. Briefly, cells were seeded in 6-well plates at 4 × 10^5^ cells/well in DMEM supplemented with 10% FBS. After a 24 h incubation, cells were approximately 50%-70% confluent, and the medium was changed to 900 μL serum-free DMEM. The lentivirus was serially diluted in Opti-MEM (Invitrogen) to obtain different MOIs from 10-100. One hundred microliters of diluted lentivirus was added to each well and incubated for 24 h. The medium was then changed, the samples were incubated for 72 h, and the puromycin-containing medium was applied to select for transduced cells.

The shRNA vector for Nanog was synthesized and purchased from Genechem Co. (Shanghai, China; Supplementary Table 3). Src siRNA was synthesized and purchased from GenePharma Co. (Shanghai, China; Supplementary Table 4). The siRNA and shRNA vectors were transfected using Lipofectamine 2000 (Invitrogen) according to the manufacturer’s instructions.

### Tumor sphere initiation assays

Cells were trypsinized and seeded in stem cell medium to evaluate self-renewal capacity by formation of tumor spheres. OS cells were harvested and seeded into 96-well plates with cell concentrations from 5 to 80 cells in 100 μL medium per well. Each well was supplemented with 20 μL of fresh medium every two days. After 14 days of culturing, culture wells with spheres were marked and spheres were counted [24].

### Western blotting

Western blotting was performed as previously described [22]. Primary antibodies included rabbit anti-human TSSC3 (1:1000; Novus biologicals), rabbit anti-human Nanog, Oct4, Sox2 (1:500; Cell Signaling Technology, Danvers, MA), rabbit anti-human Akt, p-Akt, Src, p-Src (1:1000; Cell Signaling Technology), mouse anti-human CD44 antibody (1:500; Cell Signaling Technology), mouse anti-human CXCR4 antibody (Abcam, Cambrige, MA, USA) and rabbit anti-human GAPDH (1:1000; Biodragon Immunotech, China). Secondary antibodies included an HRP-conjugated goat anti-rabbit antibody. GADPH was used as a loading control.

### Fluorescence activated cell sorting (FACS) analysis

Cells were dissociated into single cells using Accutase (Millipore, Billerica, MA), and incubated with allophycocyanin (APC) labeled anti-human CD133/CD117/Stro-1 (Miltenyi Biotec, Germany) for 30 min at 4°C. Mouse FC Block and 7-amino-actinomycin D staining (BD Falcon, Franklin Lakes, NJ) were used to eliminate nonspecific binding of antibodies and dead cells, respectively. Isotype controls were used for all experiments. The samples were washed 3 times using phosphate buffered saline (PBS), and were then analyzed by flow cytometry (FACS Aria II, BD Falcon).

### Chemo-resistance assay

One thousand cells were seeded in 96-well plates and grown to 80% confluency. The culture medium in each well was then changed to 100 μL serum-free DMEM with cisplatin at a concentration of 0, 1, 2, 4, 8, 16 or 32 μg/mL. After a 48 h incubation, the culture medium was changed to 100 μL DMEM, and 10 μL CCK-8 solution (Beyotime, Beijing) was added to each well. Cells were then incubated at 37°C for 2 h. The absorbance of each well at 450 nm was measured. The percentage of cell survival was calculated as (OD_sample-_OD_blank_)**/** (OD_0μg/ml_-OD_blank_). The dose-response curve was drawn and drug concentration required to inhibit growth by 50% (IC_50_) was calculated using SPSS 16.0 software (SPSS Inc., Chicago, IL).

### Xenografts

Subcutaneous xenograft models were generated in 4-week-old nude mice (Laboratory Animal Center, Third Military Medical University, China). Mice were randomly divided into groups and weighed every two days. Xenografts were observed and measured, and mice were weighed every two days. Xenograft-bearing mice were sacrificed and tumors were harvested and measured at the end of the 3^rd^ week. The xenografts were then fixed with formalin, sliced, and subjected to H&E staining. Animal care was performed in accordance with the guidelines of the Institutional Ethics Committee and the National Institutes of Health guide for the care and use of laboratory animals.

### Statistical analysis

All *in vitro* experiments were conducted a minimum of three times and the results presented from representative experiments. Data are expressed as the mean ± standard deviation. ELDA software was used to comapre xenograft presence (http://bioinf.wehi.edu.au/software/elda/). Other tests for statistical significance between the test and control groups were analyzed with SPSS16.0 statistical software (SPSS Inc.). When two groups were compared, the unpaired Student’s t-test was performed. When three groups were compared, one-way ANOVA analysis was performed. A non-paired sample t-test was used to compare xenograft volume. For association analysis between IHC samples and clinical-pathological parameters analysis, the Mann-Whitney U test was used for two samples and the Kruskal-Wallis test was used for three samples. *P* < 0.05 was considered statistically significant. Kaplan-Meier analysis was used for survival analysis, which was counted from the date of initial diagnosis.

## CONCLUSIONS

Our data highlights the importance of the TSSC3/Src/Akt axis in OSCs and the regulation of Nanog. TSSC3 and Nanog are important proteins that regulate OS stem cell phenotype, and Src/Akt pathway mediates regulation of Nanog by TSSC3. Therapy targeting TSSC3 and Nanog could provide a novel approach to improve prognosis.

## SUPPLEMENTARY MATERIALS FIGURES AND TABLES


