# Supplementary material for: TSSC3 represses self-renewal of osteosarcoma stem cells and Nanog expression by inhibiting the Src/Akt pathway
Source: Oncotarget. 2017 Aug 24;8(49):85628–41. doi: 10.18632/oncotarget.20429 (PMC5689636; doi:10.18632/oncotarget.20429)
Supplement: Supplementary file 1 [file oncotarget-08-85628-s001.pdf]

TSSC3 represses self-renewal of osteosarcoma stem cells and Nanog expression by inhibiting the Src/Akt pathway

SUPPLEMENTARY MATERIALS

RT-qPCR

Total RNA from OS or sarcospheres was extracted with RNAiso reagent (Takara Bio Inc., Japan). Reverse-transcription and PCR were performed using a Takara RNA PCR (AMV) kit. The sequences of each primer pair are presented in Supplementary Table 2. Real-time qPCR was performed in triplicate including non-template controls using a SYBR qPCR kit (RR820A; Takara Bio Inc.) on a real-time PCR detection system (CFX96; Bio-Rad, Hercules, CA). The cycling parameters were set up according to the manufacturer's instructions. Results were normalized to levels of the reference gene, glyceraldehyde 3-phosphate dehydrogenase (GAPDH), and the $2^{-\Delta\Delta Ct}$ method used for relative quantification.

Wound-healing assays

OS cells were seeded in 6-well plates and cultured until the cell confluency reached 90%. Two hundred microliter pipette tips were used to scratch three parallel vertical lines in every well. Wells were washed with PBS, then medium changed to serum-free DMEM. At 0 h, 12 h, 24 h, 48 h and 72 h of culture, scratch lines were imaged under a microscope, and the scratch distances were measured using ImageJ (http://rsbweb.nih.gov/ij/;NIH,Bethesda).

Transwell invasion assay

Matrigel (BD Falcon) was diluted with DMEM at ratio of 1:3 on ice. Twenty microliters of the mixture was then added in each Transwell cell culture insert (8.0 μm, Millipore), and incubated at 37ºC for 30 min. Cells were seeded in Transwell inserts at 1×10^5 cells per insert, then inserts were transferred into the wells of a 24-well plate containing 500 μL DMEM with 5 % FBS, and cultured at 37ºC. After 8 h and 24 h of culturing, inserts were washed with PBS and fixed in 4% paraformaldehyde. Cells unable to migrate were washed off, and the remaining cells were stained with crystal violet solution and counted under a microscope.

Cell stimulation

Recombinant insulin-like growth factor-1 (IGF-1, PeproTech) was added at a concentration of 2 ng/mL to culture medium after 12 h FBS-free culturing. Cells were harvested for subsequent experiments following 48 h of stimulation. LY294002 (20 µM; Selleck Chemicals, Houston, TX) or PP2 (10 µM. Selleck Chemicals) were added to culture medium for 24 h or 48 h, then cells harvested for further experimentation.

Immunofluorescence

As previously described [15], cells were cultured on glass slides in 6-well plates for 24 h. Cells were then fixed with 4% paraformaldehyde, and washed with PBS. Rabbit anti-human Nanog (1:100, Cell Signaling Technology) and Cy3-conjugated goat anti-rabbit IgG (ZSGB-bio) antibodies were used to detect Nanog expression in cells. Hoechst 33258 solution (Beyotime, Shanghai, China) was used to label cell nuclei. Cells were examined and images obtained using a laser confocal scanning microscope (LSM700, ZEISS, Germany).

Apoptosis assay

The apoptosis assay was performed using an Annexin V-APC antibody (eBioscience, Santiago, CA, UT) and 7-AAD antibody (KeyGEN, Nanjing, China). Harvested cells treated with cisplatin (Sigma-Aldrich, St. Louis, MO) for 24h at a final concentration of 3µg/ml were washed three times in PBS buffer and then resuspended in 300µl binding buffer (eBioscience). Subsequently, a total of 10µl of Annexin V-APC and 7µl of 7-AAD were added and incubated for 12min. Then the stained cells were analyzed by the flow cytometry (10,000 cells; FACS Calibur).

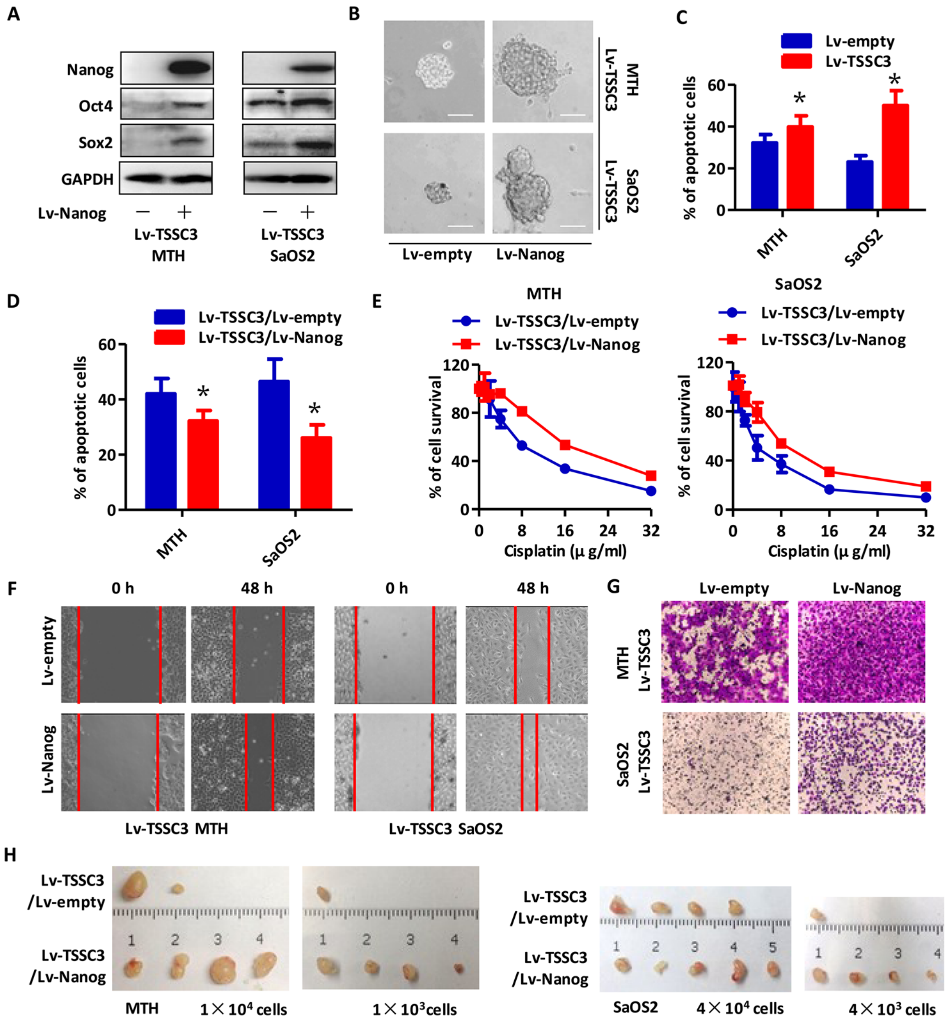

Supplementary Figure 1: Overexpression of Nanog enhances self-renewal of OS cells. **(A)** Oct4 and Sox2 expressions are elevated after Nanog overexpression in Lv-TSSC3 MTH (left) and SaOS2 (right) cells. **(B)** Tumor spheres formed by Lv-TSSC3/Lv-Nanog MTH or SaOS2 cells (right two images) are larger than those formed by Lv-TSSC3/Lv-empty cells (left two images; the scale bar represents 100 μm). **(C & D) Overexpression** of TSSC3 increases the number of apoptotic cells both in MTH and SaOS2 cells compared with Lv-empty cells; meanwhile, overexpression of Nanog decreased the number of apoptotic cells compared with Lv-TSSC3/lv-empty cells. **(E)** Overexpression of Nanog in Lv-TSSC3 MTH (left) and SaOS2 (right) cells enhances the survival following cisplatin treatment. **(F)** Migration assay for Lv-Nanog/Lv-TSSC3 MTH and SaOS2 cells. **(G)** Invasion assay for Lv-Nanog/Lv-TSSC3 MTH and SaOS2 cells. **(H)** There were fewer xenografts generated by Lv-empty/Lv-TSSC3 MTH (left) or SaOS2 (right) cells then by Lv-empty cells. Lv-Nanog/Lv-TSSC3 cells generate more xenografts.

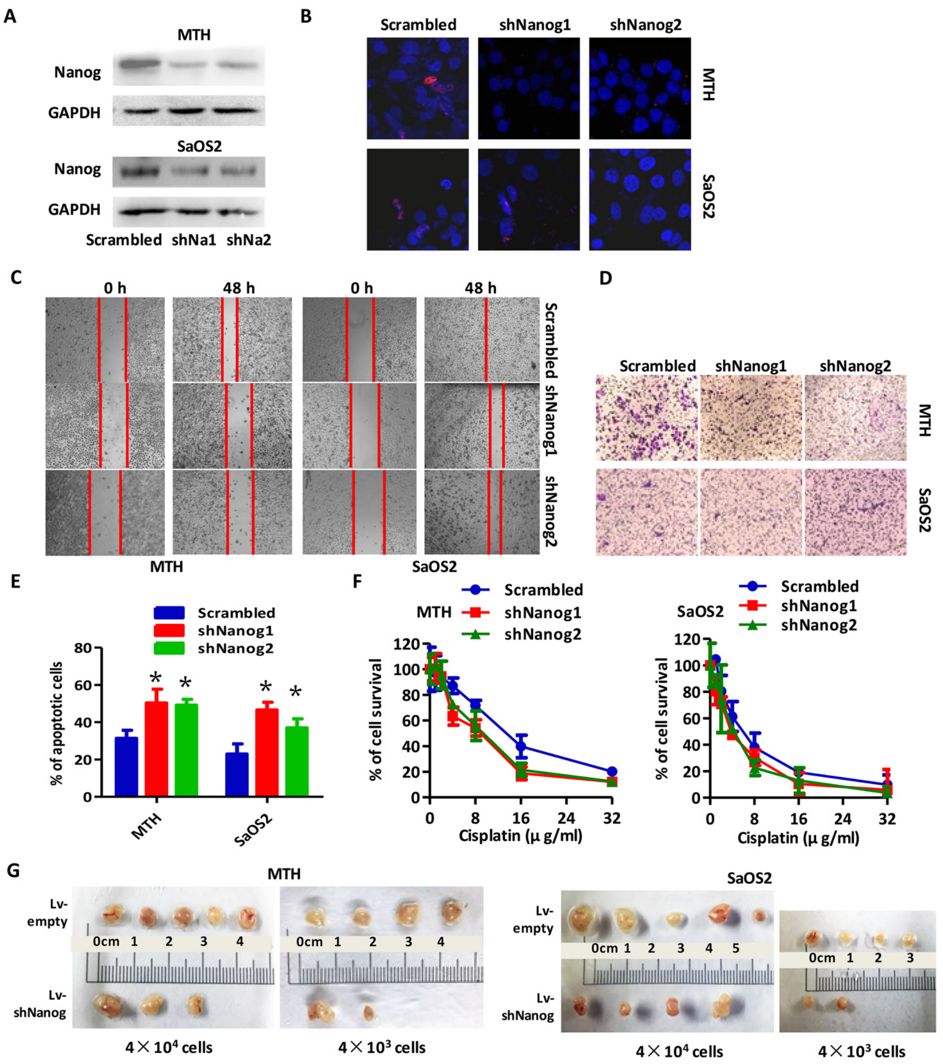

Supplementary Figure 2: Knockdown of Nanog represses a stem-like phenotype in OS cells. RT-PCR **(A)** and Western blot **(B)** of efficiency of Nanog knockdown (*$P < 0.05$). **(C)** Migration assay for shNanog MTH and SaOS2 cells. **(D)** Invasion assay for shNanog MTH and SaOS2 cells. **(E)** Knockdown of Nanog in MTH and SaOS2 cells decreased the number of apoptotic cells compared with those from scrambled cells. **(F)** Knockdown of Nanog in MTH (left) and SaOS2 (right) cells reduced the survival following cisplatin treatment compared with their respective scrambled cells. **(G)** There were fewer xenografts generated by Lv-shNanog MTH (left) or SaOS2 cells (right) then by Lv-empty cells.

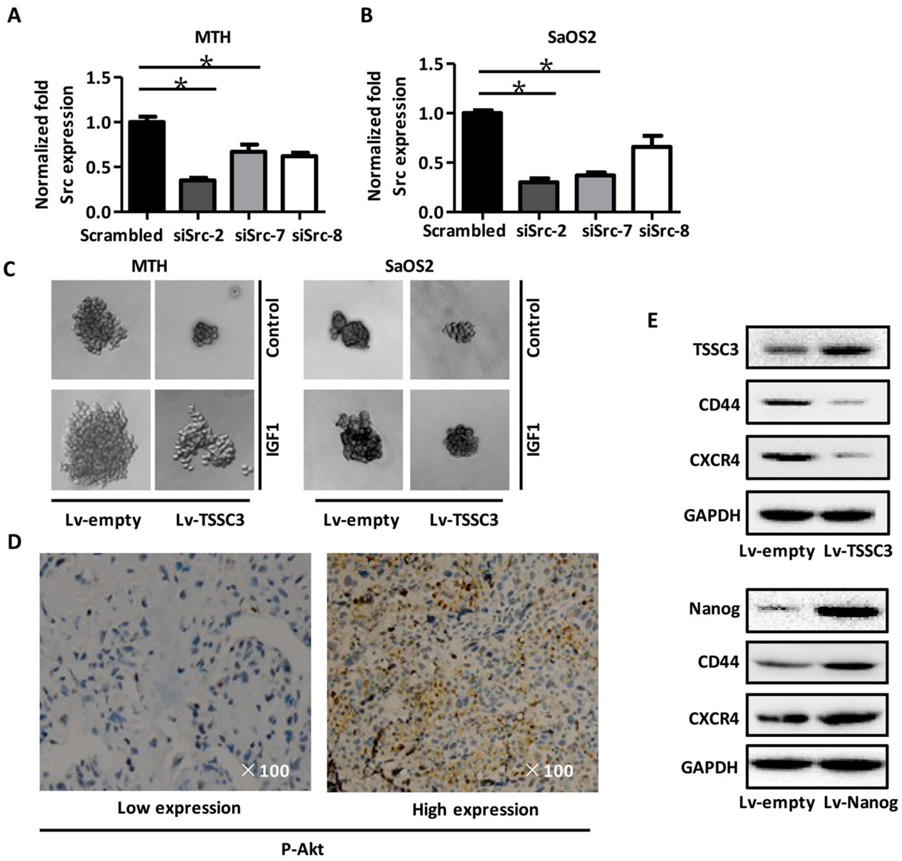

Supplementary Figure 3: The Src/Akt pathway is involved in inhibition of Nanog by TSSC3. **(A)** Efficiency of Src knockdown in MTH cells (**P* < 0.05). **(B)** Efficiency of Src knockdown in SaOS2 cells (**P* < 0.05). **(C)** Tumor spheres generated by Lv-TSSC3 MTH or SaOS2 cells are smaller than those generated by Lv-empty cells. IGF1 treatment of cells results in generation of much larger spheres. **(D)** Representative images of IHC staining for p-Akt negative (left) and positive (right) expression. **(E)** Overexpression of TSSC3 in OS cells reduced CD44 and CXCR4 expression (upper panel). Overexpression of Nanog in OS cells increased CD44 and CXCR4 expression (lower panel).

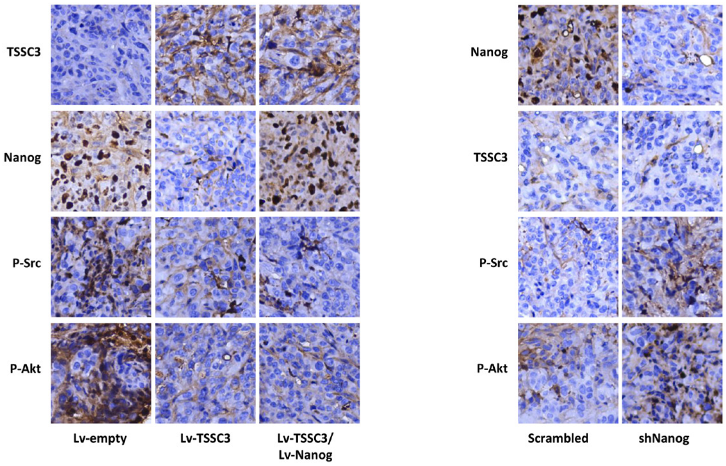

Supplementary Figure 4: IHC of TSSC3, Nanog, P-Src, and P-Akt on xenografts. (A) Overexpression of TSSC3 of OS cells generated xenografts with a reduced level of Nanog, P-Src, and P-Akt staining. Overexpression of Nanog did not impact TSSC3 expression (x100). **(B)** Knockdown of Nanog in OS cells did not alter TSSC3 expressions in xenografts (x100).

Supplementary Table 1: Clinical features of patients with osteosarcoma

Stage	No. patients	Age (years)		Gender		Tumor volume		Tumor location		
		Mean	Range	Male	Female	≥10 cm	< 10 cm	Femur	Fibula & tibia	Other
IIA	18	19.1	11-43	11	7	0	18	10	1	7
IIB	15	25.3	14-42	8	7	3	12	9	3	3
III	8	24.3	14-43	5	3	1	7	3	0	5

Supplementary Table 2: Sequences of primer pairs used in this study

Gene	Primer sequence (5'→3')	Product size
GAPDH	F: GGAGCGAGATCCCTCCAAAAT	197 bp
	R: GGCTGTTGTCATACTTCTCATGG	
Nanog	F: TTTGTGGGCCTGAAGAAAACT	116 bp
	R: AGGGCTGTCCTGAATAAGCAG	
TSSC3	F: TCCAGCTATGGAAGAAGAAGC	163 bp
	R: GTGGTGACGATGGTGAAGTACA	
SRC	F: TGGCAAGATCACCAGACGG	100 bp
	R: GGCACCTTTCGTGGTCTCAC	

Supplementary Table 3: Sequences of shRNA used in this study

	5' Stem	Loop	Stem 3'
Scrambled	TTCTCCGAACGTGTCACGT	CTCGAG	ACGTGACACGTTCGGAGAA
shNanog1	GCATGCAGTTCCAGCCAAATT	CTCGAG	AATTTGGCTGGAACTGCATGC
shNanog9	GCCGTCTCTGGCTATAGATAA	CTCGAG	TTATCTATAGCCAGAGACGGC

Supplementary Table 4: Sequences of siRNA used in this study

	Sequence (5'→3')
Srcrambled	F: UUCUCCGAACGUGUCACGUTT
	R: ACGUGACACGUUCGGAGAATT
siSrc-2	F: GCCUCAACGUGAAGCACUATT
	R: UAGUGCUUCACGUUGAGGCTT
siSrc-7	F: CAGGCUGAGGAGUGGUAUUTT
	R: AAUACCACUCCUCAGCCUGTT
siSrc-8	F: CUCGGCUCAUUGAAGACAATT
	R: UUGUCUUCAAUGAGCCGAGTT
